# Association of Breastfeeding Duration with Susceptibility to Allergy, Influenza, and Methylation Status of *TLR1* Gene

**DOI:** 10.3390/medicina55090535

**Published:** 2019-08-26

**Authors:** Ma’mon M. Hatmal, Nada N. Issa, Walhan Alshaer, Hamzeh J. Al-Ameer, Omar Abuyaman, Reema Tayyem, Nawal S. Hijjawi

**Affiliations:** 1 Department of Medical Laboratory Sciences, Faculty of Applied Health Sciences, Hashemite University, Zarqa 13133, Jordan; 2 Cell Therapy Center (CTC), The University of Jordan, Amman 11942, Jordan; 3 Department of Biology and Biotechnology, American University of Madaba, Madaba 11821, Jordan; 4 Department of Nutrition and Food Technology, Faculty of Agriculture, The University of Jordan, Amman 11942, Jordan

**Keywords:** breastfeeding, allergy, influenza, DNA methylation, *TLR1*, innate immunity, AP-1

## Abstract

*Background and Objectives:* This study aimed to investigate the possible association between exclusive breastfeeding duration during early infancy and susceptibility to allergy and influenza in adulthood. Furthermore, we also investigated the association of breastfeeding duration with DNA methylation at two sites in the promoter of the toll-like receptor-1 (*TLR1*) gene, as well as the association between DNA methylation of the toll-like receptor-1 (*TLR1*) gene and susceptibility to different diseases. *Materials and Methods:* Blood samples were collected from 100 adults and classified into two groups according to breastfeeding duration (<6 months and ≥6 months) during infancy. Subjects were asked to complete a questionnaire on their susceptibilities to different diseases and sign a consent form separately. Fifty-three samples underwent DNA extraction, and the DNA samples were divided into two aliquots, one of which was treated with bisulfite reagent. The promoter region of the *TLR1* gene was then amplified by polymerase chain reaction (PCR) and sequenced. *Results:* We found a significant association between increased breastfeeding duration and a reduction in susceptibility to influenza and allergy, as well asa significant reduction in DNA methylation within the promoter of the *TLR1* gene. No association was found between DNA methylation and susceptibility to different diseases. *Conclusions:* The findings demonstrate the significance of increased breastfeeding duration for improved health outcomes at the gene level.

## 1. Introduction

Breastfeeding has a significant impact on health, with studies reporting an association between exclusive breastfeeding duration and susceptibility to pathogenic infections and autoimmune diseases [1,2]. Breastfeeding changes the susceptibility to different diseases, which can be mediated by different epigenetic factors [3,4,5,6,7]. Results from previous studies showed lower morbidity and mortality rates among breastfed infants compared to non-breastfeed ones [8]. Human milk has been found to play an important role in the regulation of human genes through epigenetic modulators, and the period in which an individual is most vulnerable to epigenetic changes is before the age of two years [4]. Recent studies have demonstrated that miRNAs control the expression of key epigenetic modulators, including DNA methyltransferases and histone deacetylases [9,10,11,12,13,14].

DNA methylation involves the addition of a methyl group to the 5’ position of thecytosine ring, and nearly 75% of all CpGs (C nucleotide followed by G nucleotide in the same strand) are methylated in mammals. It is proposed that breastfeeding could affect DNA methylation of the human genome due to the presence of miRNA-148a-3p and miRNA-146b-5p [15]. One of the most important functions of 148a–3p is its interference with the function of DNA methyltransferase 3b (DNMT3b), which is responsible for de novo methylation during the embryonic stage of fetal development and maintaining the methylation of DNA via DNA methyltransferase-1 (DNMT1) [15,16]. A knockout of DNMT3b in mice causes lymphomagenesis, due to demethylation in normal thymocytes [17].

The family of toll-like receptor (*TLR*) genes are targets for nuclear methylation modification, and play significant roles in innate immunity [18]. This group includes many receptors, from TLR1 to TLR13 [18,19,20]. TLR1 is ubiquitously expressed, with higher levels being present in B-lymphocytes [20]. Human milk also participates in the inhibition of the TLR signaling pathway of the intestinal epithelia, and reduces the incidence of enteric inflammation [21]. The presence of TLR regulatory components within human milk provides a benign oral prophylactic for many inflammatory disorders mediated by abnormal TLR signaling, such as inflammatory bowel disease [22]. Extensive investigation in this field of research may provide results which can be used to manufacture additives for milk formulas to obtain similar effects in the future.

The current study examines the impact of breastfeeding duration on DNA methylation of the *TLR1* gene through an investigation of the DNA methylation of two CpG islands located within or close to the binding sites of the transcription factor activated protein-1 (AP-1). Transcription factor AP-1 is involved in cellular proliferation and transformation [23,24], and binding of AP-1 can be affected by DNA methylation [25]. It has been shown that both AP-1 binding sites (Tables S1 and S2) are close to each other and important to AP-1 binding in the*TLR1* gene. Throughout the formation of different binding complexes involving c-Jun, c-Fos, and AP-1 proteins, these two sites are either competing or coinciding with each other based on different binding conditions, and they play mutual roles in regulating *TLR1* gene expression [26]. The results of one study proved that the first region (in which the first CG island is located) serves as a docking area for two complexes that contain ATF-2 plus JunD and c-Jun plus c-Fos. Overall, the existence of functional AP-1 binding sites in the proximal promoter region of theTLR1 promoter was verified, and some of them are involved in the constitutive expression of the *TLR1* gene [26]. It was also reported that the expression level of some genes correlates with the hypomethylation of CpG islands within the context of AP-1 binding sites of different genes [26,27]. For example, it was found that the DNA-methylation-dependent AP-1 binding site was functionally active in the *mts1* gene. When methylated, this site reproducibly repressed the transcription of CAT-containing DNA that had been transiently transfected into mouse adenocarcinoma CSML100 cells [27]. It was also found that the8-bp-long binding site containing a CpG is affected by CpG methylation [27]. Furthermore, it was reported that AP-1 interactions with DNA can be inhibited by methylating the CpGs adjacent to their respective DNA binding sites. Similarly, methylation adjacent to the core Sp1 motif induces a significant decrease in Sp1/Sp3 binding [28]. Interestingly, this phenomenon also occurs in an allele-specific manner. For instance, YY1 binding events are modulated by DNA methylation in a parent-of-origin-specific fashion, in such a way that only the CpGs close to the binding site of the maternal allele are methylated, preventing YY1 from binding only the maternal allele [28]. These studies show the importance of the DNA methylation of CpG islands to the binding of AP-1 and other proteins, which then affects the expression of the corresponding genes [28].

Breastfeeding for the first six months of life, without any external supplements, is highly recommended by the WHO [29], and it has been indicated that breastfeeding can improve human health. Breastfeeding modulates the expression of human genes, specifically those involved in the immune system [21]. The main aim of the present study was to determine the effects of increased breastfeeding duration on the susceptibility to influenza and different allergies (i.e., asthma, food allergies, conjunctivitis, hay fever, and eczema).For allergies and influenza, an association between different allergies and different types of respiratory infections has previously been reported. For example, an association was reported between food allergy and allergic respiratory disease with recurrent respiratory tract infections during childhood [30,31]. Moreover, other studies have reported that respiratory infections (i.e., viral infections) promote allergic sensitization and asthma in animal models [32,33]. In addition, Holt et al. provided evidence that viral infections in early life trigger the development of asthma [34]. This has recently been proven by the findings of the critical role of IL-25 and IL-33 after rhinovirus infections and the development of asthma. A new term, “The viral march”, was even proposed to describe the connection between early life virus wheezing and subsequent asthma. Furthermore, the treatment of allergic diseases, such as the use of allergen-specific immunotherapy, can reduce the incidence of respiratory infections [34].

The association of breastfeeding duration with long-term DNA demethylation at two sites in the promoter of the toll-like receptor-1 (*TLR1*) gene was determined in this study. Furthermore, analysis to find the association between DNA methylation of the promoter of *TLR1* and susceptibility to allergy and influenza was investigated. We hypothesize that exclusive breastfeeding reduces DNA methylation of the promoter of the *TLR1* gene thereby activating *TLR1* expression, and thus could improve immunity and disease outcomes.

## 2. Materials and Methods

### 2.1. Blood Samples Collection and Clinical History of Volunteers

This was a pilot cross-sectional study that took place from November 2015 until August 2018 at the Hashemite University. The study protocol was designed according to the ethical guidelines of the 1975 Declaration of Helsinki, and the study was approved by the Institutional Review Board at Hashemite University (no. 2015/2014/3/5) (date: 25/3/2015). Informed consent was obtained from the participants. All information collected was treated with complete confidentiality and the data were used only for this research purpose. There were no associated risks due to participation in this research project.

Questionnaires were completed by 100 volunteers (aged between 18 and 25 years old) and their mothers in order to collect clinical (i.e., disease susceptibility) and para-clinical history data (Tables S1 and S2). The selection was random, and in order to reduce the recall bias by the volunteers, a phone interview was then done with their mothers to clarify information like breastfeeding duration and susceptibility to different diseases.

Blood samples from the volunteers were collected (after signing a consent form) and classified into two groups: group A received exclusive breastfeeding <6 months and group B for ≥6 months. This classification was done according to WHO 2014 recommendations for infant nutrition [29]. Group A contained 55 samples (23 male, 32 female) from volunteers who had been exclusively breastfed for <6 months, while Group B contained 45 samples (16 male, 29 female) from volunteers who had been exclusively breastfed for ≥6 months (no significant difference was found in gender distribution between the two groups). Limitations such as recall bias of the breastfeeding mothers was still found, but the effect was reduced by checking the start and end dates of breastfeeding with mothers, and by grouping breastfeeding duration into two groups (<6 months and ≥6 months), thus any recall bias was significantly reduced unless it was on the borderline. For the borderline people, we checked the data again with the mothers. Recall bias regarding other information (i.e., susceptibility to different diseases) was reduced by asking the same questions for the volunteers and their mothers individually, and by using different ways of asking to ensure they understood the questions. For only those involved in DNA methylation analysis, the mean, median, and standard deviation for group A were 3.1, 4.0, and 1.58 months, and for group B they were 9.4, 7.0, and 4.3 months, respectively. Moreover, *t*-test and Wilcoxon test were performed between group A and group B to check if there was a difference in breastfeeding duration between the two groups. A *p*-value <0.05 indicated a significant difference in the breastfeeding duration between the two groups.

### 2.2. Criteria for Defining Different Diseases

The criteria for diagnosing influenza were based on the duration of symptoms as defined by the CDC [30]. Participants who had been infected with influenza more than twice per winter season and had suffered from severe symptoms were considered positive, while others who did not meet the criteria were considered negative [35]. For allergy, volunteers were simply self-reported based on medical examination history.

### 2.3. Naïve B Cell Isolation

Blood samples were collected from each volunteer in duplicate in 2 mL EDTA tubes and then diluted 1:1 in 1× PBS. Each sample was then layered over 4 mL of Ficoll-Paque Plus in a 15 mL Falcon tube and subjected to centrifugation at 400× *g* for 40 min. The lymphocyte layer was transferred from each tube individually using a sterile Pasteur pipette into a fresh 15 mL Falcon tube and subjected to centrifugation at 400× *g* for 10 min. The pellet was collected, and naïve B cell isolation was performed using a B cell isolation kit II from Miltenyi Biotic Inc (Bergisch, Gladbach, Germany). The isolated lymphocytes were resuspended in 40 µL of 1× PBS containing 0.5% BSA and then incubated at 4 °C in 10 µL of Naïve B cell Biotin-Antibody Cocktail against CD2, CD14, CD16, CD27, CD36, CD43, and CD235a (Glycophorin A). Next, 30 µL from the prepared 1× PBS buffer was added to the cells, followed by incubation with 20 µL of anti-biotin microbeads for 10 min at 4 °C to immobilize all cells other than naïve B cells. To separate the naïve B cells, a magnetic-based separation column was employed. A magnetic column was connected to a magnetic separator and prepared by loading 500 µL of 1× PBS onto it and then removing the eluted buffer. Each sample was then individually loaded onto the magnetic column and the elution of the B cells was accomplished using a negative selection method into a sterile 1.5 mLtube. Finally, 500 µL of 1× PBS was added for a second elution buffer to ensure that all the targeted B cells had been eluted. The eluted cells were subjected to centrifugation at 800× *g* for 5 min at 4 °C, and the supernatant was removed while the sedimented cells were stored for further DNA extraction. The whole procedure for the separation and isolation of cells was performed under sterile conditions.

### 2.4. DNA Extraction and Bisulfite Treatment

DNA extraction from naïve B lymphocytes was performed using a QIAamp®DNA Blood Mini Kit (Qiagen, Hilden, Germany) according to the manufacturer’s instructions. Briefly, isolated naïve B cells were lysed by adding 20 µL of protease solvent and 200 µL of AL buffer (lysis buffer), and mixed by vortexing for 15 s, before being incubated at 56 °C for 10 min. Next, 200 µL of absolute ethanol was added to the cell lysate and mixed by vortexing for 15 s. The mixture was then transferred to a spin column which was provided in the kit and subjected to centrifugation at 8000 RPM for 1 min. The filtered solution was discarded, and the spin column was transferred to a new collection tube, before adding 500 µL of AW1 (washing buffer 1) to the spin column and repeating the centrifugation step. This was followed by adding 500 µL of AW2 (washing buffer 2) to the spin column and subjecting it to centrifugation at 14,000 RPM for 3 min. The filtered solution was again discarded, and the spin column was subjected to centrifugation at 14,000 RPM for 1 min. The spin column was transferred to a sterile 1.5-mL microfuge tube and 70–100 µL of AE buffer (elution buffer) was added to the spin column, which was incubated at room temperature for 30 min before eluting the DNA extracted from the B cells by centrifugation at 8000 RPM for 1 min. The concentration and purity of the extracted DNA were measured using a Thermo Fisher Scientific NanoDrop 2000C spectrophotometer (Thermo Fisher, Waltham, MA, USA).

The extracted DNA from each sample was divided into two aliquots. The first was used in the polymerase chain reaction (PCR) amplification of the *TLR1* proximal promoter, and the second was treated with bisulfite reagent (which deaminates unmethylated cytosine into uracil, while keeping methylated cytosine unaffected) according to the manufacturer’s instructions for the EZ DNA Methylation-Lightning™ Kit (Zymoresearch, Irvine, CA, USA). Bisulfite treatment commenced by adding 130 µL of lightning conversion reagent to 20 µL of DNA, and the treated DNA was then incubated at 98 °C for 8 min, and then at 54 °C for 60 min. The incubated DNA was then transferred to a Zymo-Spin™IC Column (Zymoresearch, Irvine, USA) which was previously loaded with 600 µL of M-binding buffer and mixed by inverting the spin column several times. The column was subjected to centrifugation at maximum speed (~10,000× *g*) for 30 s. The flow-through was discarded, 100 µL of M-washing buffer was added to the spin column, and the centrifugation step was repeated. After washing the DNA, 200 µL of L-Desulphonation buffer was added to the spin column, which was incubated at room temperature for 20–30 min and the centrifugation step was again repeated, followed by washing twice with 200 µL of M-washing buffer. Finally, the treated DNA was eluted using 10 µL of M-elution buffer.

### 2.5. PCR Amplification of the TLR1 Promoter Before and After Bisulfate Treatment

The *TLR1* proximal promoter was amplified in a 25 µL total volume PCR reaction mix. For DNA prior to bisulfite treatment, the forward primer was 5’- TCCATCCTGTAACCAGCACA-3’and the reverse primer 5’- TCTGTGGGTTACCTGATAGGC-3’. The PCR program was as follows: initial denaturation at 95 °C for 3 min, then 45 cycles of 30 s at 95 °C, 40 s at 53.3 °C, and 50 s at 72 °C, followed by a final extension at 72 °C for 10 min. For DNA that had undergone bisulfite treatment, the forward primer was 5’-GAAAAGATTAGTGGAAAAAAATTTAG-3’and the reverse primer 5’-TCCAAATTTCACTACAATCAAT-3’. The PCR program was as follows: initial denaturation at 95 °C for 2 min, then 45 cycles of 30 s at 95 °C, 40 s at 53.3 °C, and 50 s at 72 °C, followed by a final extension at 72 °C for 10 min. The PCR products from all the amplification reactions were separated by 1.7% agarose gel electrophoresis and visualized using a gel documentation system.

### 2.6. DNA Sequencing and Analysis

The DNA sequencing was performed for 53 samples before and after bisulfite treatment (by Macrogen Humanizing Genomics Company (Seoul, Korea)) based on the modified Sanger method (dye chain-termination). The samples for DNA sequencing were selected because they contained enough DNA for sequencing; other samples were determined to contain low DNA quantities unsuitable for DNA sequencing.

### 2.7. Statistical Analysis

SPSS version 20.0 software (SPSS Inc., Chicago, IL, USA) was used to perform the statistical analysis. Chi-square test (Yates continuity correction) with a significance level of 0.05 was employed in the data analysis.

## 3. Results

### 3.1. Association of Breastfeeding with Susceptibility to Influenza and Allergies

In this study, 100 volunteers were classified into two groups: group A received exclusive breastfeeding <6 months, and group B for ≥6 months. All the volunteers were surveyed for their susceptibility to influenza and allergies. The criteria for the diagnosis of influenza were dependent on the duration of influenza symptoms during the winter season, and participants who had been infected with influenza >2 times with severe symptoms were considered positive, while others were negative. Allergy was verified according to whether a volunteer had experienced the diseases or not based on medical examination history.

Table 1 shows the Chi-square test results for the association between the duration of breastfeeding and volunteers being affected by influenza or allergies. The total number of people affected by influenza or allergies were 27 and 24, respectively. The table also shows the number of males and females for each disease. Group A is the group which received breastfeeding less than 6 months (average breastfeeding was 3.1 months), while group B received breastfeeding more than or equal to 6 months (average breastfeeding 9.4 months). The *p-*value for all “total” and “female” sections was <0.05.

### 3.2. DNA Methylation at AP-1Binding Sites

**Figure 1:**
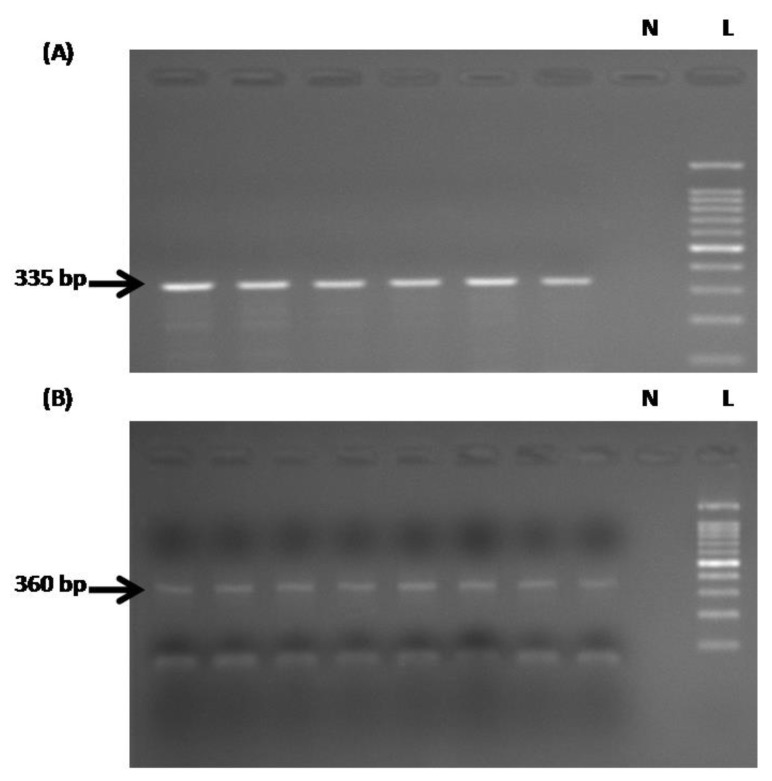
Gel electrophoresis analysis of PCR amplification of the target region of the *TLR1* promoter region. (**A**) DNA prior to bisulfite treatment, and (**B**) DNA after bisulfite treatment. The different product sizes are due to the different primers used. “L” indicates100-bp DNA ladder, and “N” indicates negative control.

Agarose gel electrophoresis analysis of PCR amplification products from DNA prior to bisulfite treatment (Figure 1A) revealed a band which was approximately 335 bp in size, whereas it was 360 bp in size after bisulfite treatment (Figure 1B). The difference in size was due to the usage of different primer sets before and after bisulfite analysis. Only samples with sufficient DNA quantity were sent for DNA sequencing.

DNA methylation status at two CpG islands of the AP-1 binding site and its association with susceptibility to different diseases are shown in Table 2, while DNA methylation status at the two CpG islands of AP-1 binding sites and its association with the breastfeeding duration are shown in Table S3. We provide the methylation status for each region in Table S1 for group A and Table S2 for group B. In the statistical analysis, we combined both regions because they were reported to be in or near the putative binding site of the AP-1 protein. These regions are in close proximity to each other, and it has been shown that both regions are important to AP-1 binding in *TLR1* gene by the formation of different binding complexes. These regions were shown by other studies to play mutual roles in binding with different proteins, including AP-1 [26].

The *p*-value for the Chi-square test was >0.05. The detailed inspection of individuals in group A and group B are shown in Tables S1,S2 (where they show DNA methylation, duration of breastfeeding, and susceptibility to different diseases for volunteers whose samples were sequenced). People with less than 6 months of breastfeeding were distributed equally between “Yes” and “No” groups of the methylation states. It was noticed that people with 6 months or more of breastfeeding were mainly distributed among “No” status (which indicates that at least one of the two CpG islands was unmethylated), thus providing a chance for the binding complexes to bind and stimulate *TLR1*.

## 4. Discussion

The association between breastfeeding duration and decreased susceptibility to different diseases has previously been reported [36]. The present study was targeted to determine the effects of increased breastfeeding duration on the susceptibility to different diseases (i.e., allergies and influenza) among adults. Additionally, the association of breastfeeding duration with DNA methylation at two sites in the promoter of the*TLR1* gene was investigated; these two sites were identified to be within or adjacent to AP-1 binding sites, and their methylation statuses was determined to affect the binding of AP-1 and other proteins [28].

The results in Table 1 show a significant reduction in the susceptibility to influenza in group B (≥6 months of breastfeeding) compared to group A (<6 months of breastfeeding) (*p* < 0.05). These results agree with the study by Ip et al. [37], which reaffirms the recommendation for exclusive breastfeeding for approximately the first 6 months of life, followed by complementation with food supplements for a year or longer. The study by Ip et al. [37] showed a significant reduction in upper and lower respiratory tract infections in young children of up to 77% among 400 individuals. This finding may support the role of other members of the TLR family, such as TLR3, TLR4, TLR7, TLR8, and TLR9, which promote Th1 responses against intracellular pathogens directed by cell-mediated immunity, and induce B cells to release high levels of IgG and IgA antibodies that drive phagocytic activation and defense against intracellular pathogens such as influenza [20].

Breastmilk contains important immune-modulators, such as TLR agonists and antagonists, in addition to high concentrations of transforming growth factor-beta (TGF-β) and IL10, which are inducers for Foxp3 (+) CD25 (+) CD4 (+) regulatory T cell (T-reg) production, which act as anti-inflammatory regulators [20,29]. This indicates that breast milk has a significant role in inducing attacks against pathogens, while concurrently inducing the infant body to shift the immune system to the formation of T-reg memory cells, which occurs following a second exposure to a pathogen, thereby maintaining T cell tolerance activation and reducing the symptoms of influenza infection [38].

Table 1 also reveals a significant association between increased breastfeeding duration and decreased susceptibility (particularly in females) to different types of allergies (i.e., asthma, food allergies, conjunctivitis, hay fever, and eczema), with a *p*-value <0.05. These allergies were confirmed based on medical examination history of the volunteers. Females are more susceptible to allergies than males due to differences in sex hormones and due to pregnancy [39,40,41,42,43], which might produce profound differences based on breastfeeding duration. This effect wasnot observed when only the patients selected for methylation analysis were included, which might be attributed to the decrease in sample size. In the study by Ip et al., it was shown that breastfeeding ≥3 months was associated with a significant reduction in the incidence of asthma and atopic dermatitis (44% and 42%, respectively)in children with a familial history, and a reduction of 26% and 27%, respectively, in children without a familial history. Greer et al [44] showed that more research is needed to prove the effect of delaying the time of the introduction of complementary foods to beyond 4 to 6 months of age in relation to the occurrence of atopic disease, while the American Academy of Pediatrics at the end of 2008 found that for infants at high risk of atopic diseases there is evidence to support that exclusive breastfeeding for at least 4 months, and delaying complementary foods until 4–6 months, prevents the development of allergies [45]. However, an expert panel sponsored by the National Institute of Allergy and Infectious Diseases (NIAID) has recently recommended that peanut-containing foods should be introduced into infants’ diets as early as 4 to 6 months of age to decrease the risk of developing peanut allergy [46].

DNA methylation is one of the most stable DNA epigenetic regulations that can alter gene expression during embryonic and adult life stages. DNA methylation is typically removed during zygote formation and re-established through successive cell divisions during development [4]. Hyper-methylation or hypo-methylation determines whether a gene is highly expressed or silenced [47].

In our study, the *TLR1* promoter region was sequenced before and after bisulfite treatment (a classical method which provides qualitative data regarding DNA methylation, still considered as one of the main methods in revealing a more sophisticated role of DNA methylation [28]) to determine if there was a relationship between breastfeeding duration and the DNA methylation pattern at two CpG sites within the promoter region of *TLR1* close to AP-1 binding sites. The Chi-square test (Yates-corrected) results presented in Table S3 show that there was a significant reduction in methylation for at least one CpG island in close proximity to an AP-1 binding site in volunteers who had been breastfed ≥6 months, with a *p*-value <0.05 (detailed results are presented in Tables S1,S2).

Human breast milk is considered to be a rich source of miR-148a and miR-146b, which enter an infant’s circulation and can interrupt epigenetic modifications (methylation) of human DNA by interfering with some nuclear DNMTases, such as DNAMT3b expression and the NF-κB signaling pathway of TLRs, respectively [9,11,15]. The *TLR1* gene is highly expressed in B lymphocytes, and could be affected by interference of this DNA methylation [48].

Results of the Chi-square test (Yates corrected) also indicated that there was no association between DNA methylation of the promoter of the *TLR1*and susceptibility to either allergies or influenza (Table 2). The association of breastfeeding duration (not DNA methylation of the promoter of *TLR1*) with susceptibility to influenza indicates that breastfeeding may have multiple mechanisms other than the methylation of *TLR1* to reduce the susceptibility to both allergy and influenza. This agrees with a previous study showing that *TLR1* (i.e., N248S variants) had no association with atopic diseases [49]; it was even correlated with less total IgE levels, which indicates that interaction with other genetic and environmental factors might be required to contribute to atopic and allergic diseases [49].

The findings of our study provide a clue for further studies to investigate the exact mechanism of the association between breastfeeding duration and methylation of the*TLR1* gene. We suggest that future studies increase the sample size and include confounding factors (i.e., vaccination status, antiviral/antibiotic usage, severity of allergies, microbiome, and nutritional status).

## 5. Conclusions

This study found a significant association between increased breastfeeding duration and susceptibility to influenza and allergies. Among the 53 DNA samples that were sequenced and analyzed before and after bisulfite treatment, a significant reduction in the DNA methylation of two CpG islands within the *TLR1* gene promoter region and close to binding sites for the AP-1 transcription factor was observed. These findings indicate the significant influence of increased breastfeeding duration on genes involved in human immunity and on the overall improvements of health outcomes. In the future, determining the association of breastfeeding with the demethylation of new DNA sites in the promoter region of *TLR1*could explain some of the immune modulations affected by breastfeeding.

## Figures and Tables

**Table 1:** Chi-square test results for the association between the duration of breastfeeding and volunteers being either affected or not affected by one or more chronic non-communicable disease (i.e., influenza or allergies).

Disease	Category	<6 months		≥6 months		*p*-Value
		Yes	No	Yes	No	
Influenza	Total	27 (20)	28 (8)	6 (6)	39 (19)	0.00 (0.00)
	Males	13	10	3	13	0.04
	Females	14	18	3	26	0.01
Allergies	Total	24 (10)	31 (18)	9 (4)	36 (21)	0.02 (0.12)
	Males	9	14	3	13	0.31
	Females	15	17	5	24	0.03

Total: both males and females. Yes: volunteers suffered from this disease. No: volunteers did not suffer from this disease. Numbers in brackets correspond only to the patients involved in DNA methylation.

**Table 2:** Chi-square test (Yates-corrected) results for the association between different diseases and the methylation of the two CpG sites within the *TLR1* gene promoter region in close proximity to the AP-1 binding site.

Disease	Methylation at the Two Sites Together		Unmethylation of Least One Site		*p*-Value
	Yes	No	Yes	No	
Influenza	12	5	13	18	0.08
Allergies	6	11	7	24	0.50

“Yes” indicates that the patient had the disease, while “No” indicates that the patient did not have the disease.

## Supplementary Materials

Supplementary Materials can be found at https://www.mdpi.com/1010-660X/55/9/535/s1. Table S1: Methylation status of the two CpG islands (GACGTGATTAA and TTTACGAGTG) in the TLR1 promoter region for the volunteers who were breastfed for <6 months (Group A). Table S2: Methylation status of the two CpG islands (GACGTGATTAA and TTTACGAGTG) in the TLR1 promoter region for the volunteers who were breastfed for ≥6 months (Group B). Table S3: Chi-square test (Yates-corrected) results for the association between the duration of breastfeeding and methylation of the two CpG sites within the TLR1 gene promoter region in close proximity to the AP-1 binding site.
