# Supplementary material for: Association of Breastfeeding Duration with Susceptibility to Allergy, Influenza, and Methylation Status of TLR1 Gene
Source: Medicina (Kaunas). 2019 Aug 26;55(9):535. doi: 10.3390/medicina55090535 (PMC6780093; doi:10.3390/medicina55090535)
Supplement: Supplementary file 1 [file medicina-55-00535-s001.pdf]

Table S1. Methylation status of the two CpG islands (GACGTGATTAA and TTTACGAGTG) in the *TLR1* promoter region for the volunteers who were breastfed for <6 months (Group A).

Sample ID	Duration of Breastfeeding (months)	GACGTGATTA[a]	TTTACGAGTG[a]	Methylation status at the two sites together	Allergies[b]	Influenza[b]
1	4	+	+	Yes	+	+
2	3	N	-	No	-	+
3	4	N	-	No	+	+
4	4	+	+	Yes	+	-
5	0.5	N	N	N/A	+	+
6	3	+	-	No	+	+
7	4	+	+	Yes	-	+
8	2	+	-	No	-	+
9	3	N	-	No	+	+
10	4	+	+	Yes	-	+
11	2	-	N	No	-	+
12	0	+	+	Yes	-	-
13	0	+	-	No	+	+
14	0.5	+	+	Yes	-	+
15	4	+	+	Yes	+	+
16	0	N	-	No	-	+
17	5	+	+	Yes	-	+
18	4	-	N	No	-	-
19	3	-	N	No	-	+
20	4	+	+	Yes	-	+
21	4	N	-	No	-	-
22	5	+	+	Yes	+	-
23	2	+	+	Yes	-	+
24	4	+	+	Yes	-	+
25	5	+	+	Yes	+	-
26	4	+	+	Yes	-	+
27	4	N	-	No	-	-
28	4	N	+	N/A	-	-

[a] N: Not determined. Yes: both sites were methylated; No: at least one site was non-methylated; N/A: the status was not available. [b] +: Indicates the susceptibility to the corresponding disease.

Table S2. Methylation status of the two CpG islands (GACGTGATTAA and TTTACGAGTG) in the *TLR1* promoter region for the volunteers who were breastfed for ≥6 months (Group B).

Sample ID	Duration of Breastfeeding (months)	GACGTGATTA[a]	TTTACGAGTG[a]	Methylation status at the two sites together	Allergies[b]	Influenza[b]
1	6	+	-	No	-	-
2	10	N	+	N/A	-	-
3	11	+	-	No	-	-
4	18	-	N	No	+	+
5	9	+	-	No	-	-
6	7	+	-	No	-	-
7	6	-	+	No	-	+
8	18	N	-	No	-	+
9	7	-	+	No	-	-
10	8	-	N	No	-	-
11	6	+	-	No	-	-
12	12	+	-	No	+	-
13	12	N	N	N/A	-	-
14	12	N	-	No	-	-
15	8	N	-	No	-	-
16	6	-	N	No	-	-
17	6	-	N	No	-	-
18	6	-	N	No	-	-
19	18	-	-	No	-	-
20	6	+	+	Yes	-	-
21	7	+	+	Yes	-	+
22	6	+	-	No	-	-
23	18	-	+	No	+	+
24	6	N	+	N/A	-	-
25	6	+	+	Yes	+	+

[a] N: Not determined. Yes: both sites were methylated; No: at least one site was non-methylated; N/A: the status was not available. [b] +: Indicates the susceptibility to the corresponding disease.

Table S3. Chi-square test (Yates-corrected) results for the association between the duration of breastfeeding and methylation of the two CpG sites within the *TLR1* gene promoter region in close proximity to the AP-1 binding site.

Group A Methylation at the two sites together		Group B Un-methylation at at least one site		*p*-value
Yes	No	Yes	No	
14	12	3	19	0.005

"Yes" indicates that both sites were methylated, while "NO" indicates that at least one site was unmethylated.
